# Influence of Silicodactyly in the Preparation of Hybrid Materials

**DOI:** 10.3390/molecules24050848

**Published:** 2019-02-28

**Authors:** Chiara Ivaldi, Ivana Miletto, Geo Paul, Giovanni B. Giovenzana, Alberto Fraccarollo, Maurizio Cossi, Leonardo Marchese, Enrica Gianotti

**Affiliations:** 1 Dipartimento di Scienze e Innovazione Tecnologica, Università del Piemonte Orientale, V. T. Michel 11, I-15100 Alessandria, Italy; chiara.ivaldi@uniupo.it (C.I.); ivana.miletto@uniupo.it (I.M.); geo.paul@uniupo.it (G.P.); alberto.fraccarollo@uniupo.it (A.F.); maurizio.cossi@uniupo.it (M.C.); leonardo.marchese@uniupo.it (L.M.); 2 Dipartimento di Scienze del Farmaco, Università del Piemonte Orientale, Largo Donegani 2/3, I-28100 Novara, Italy; giovannibattista.giovenzana@uniupo.it; 3 CAGE Chemicals srl, Via Bovio 6, I-28100 Novara, Italy

**Keywords:** hybrid materials, silicodactyly, physico-chemical characterization

## Abstract

The organic–inorganic hybrid materials have attracted great attention due to their improved or unusual properties that open promising applications in different areas such as optics, electronics, energy, environment, biology, medicine and heterogeneous catalysis. Different types of silicodactyl platforms grafted on silica inorganic supports can be used to synthesize hybrid materials. A careful evaluation of the dactyly of the organic precursors, normally alkoxysilanes, and of the type of interaction with the inorganic supports is presented. In fact, depending on the hydrophilicity of the silica surface (e.g., number and density of surface silanols) as well as on the grafting conditions, the hydrolysis and condensation reaction of the silylated moieties can involve only one or two out of three alkoxysilane groups. The influence of silicodactyly in the preparation of organic-inorganic silica-based hybrids is studied by TGA, ^29^Si, ^1^H and ^13^C solid-state NMR and FTIR spectroscopies, with the support of Molecular Dynamics calculations. Computational studies are used to forecast the influence of the different grafting configurations on the tendency of the silane to stick on the inorganic surface.

## 1. Introduction

In the recent past, great progress in the ability of designing new organic-inorganic hybrid materials, with tuned properties, has been made [1,2,3,4,5]. The possibility of functionalizing inorganic supports with organic moieties combining the enormous functional variation of organic chemistry with a high thermally and mechanically stable inorganic matrix, has paved the way for a large number of applications in many fields of chemistry, such as optics, electronics, energy, environment, biology, medicine and heterogeneous catalysis [6,7,8]. Among hybrid silica-based materials, great attention has been focused on ordered mesoporous silicas such as MCM-41 to be used as inorganic support, due to their large specific surface areas and tunable ordered pore arrays with controlled size. These mesoporous systems offer huge porous volumes and the possibility of easy surface functionalization (e.g., by post-synthetic grafting) [9], suitable for the molecular capture, catalytic reactions, enzyme immobilization [10] and drug delivery [11,12]. The MCM-41 functionalization not only enables the decoration of the MCM-41 with a wide variety of organic groups but also ensures a good dispersion of the organic moieties inside the internal surface of the support, minimizing the aggregation of the active sites and avoiding their migration. In particular, organic-inorganic hybrid catalysts exhibited excellent catalytic activity and reusability, as a result of a better embedment of the active sites, in comparison with traditional physical entrapment methods [13]. Despite the importance of hybrid silica-based materials in heterogeneous catalysis, the factors influencing the conformation of the organic moiety grafted on the surface have not been systematically studied. In particular, the best catalytic performances are expected when the organic moiety is able to stay far from the surface, thus fully available for interaction with substrates. On the other hand, the degree of hydrolysis and condensation during the grafting procedure, the hydrophilicity (i.e., density of silanols) of the silica surface, the confinement effects, as well as the length of the anchoring groups may lead the organic moiety to lay on the surface, possibly hampering the catalytic activity.

**Scheme 1:**
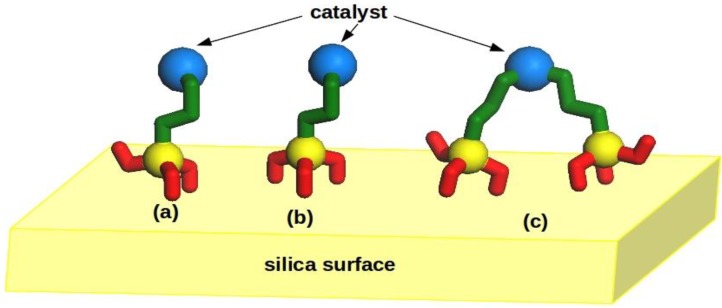
Examples of different silicodactyly (**a**,**b**) or silicopodality (**c**), influencing the conformation of the organic moiety with respect to the surface. Yellow sphere: Silicon; red: Hydrolysable tails; green: Alkyl chain; blue: Generic organic catalytic active sites.

This study aims to investigate how the architecture of organosilanes grafted on a silica surface influences the conformation of the organic chains: Two important parameters can be easily varied during the synthesis, as sketched in Scheme 1.

First, each alkoxysilane can bear 1 to 3 hydrolysable tails (typically methoxy or ethoxy groups), which can react with surface silanols to form siloxane bridges: The number of such bridges actually formed depends on the number of hydrolysable groups and on the concentration of surface silanols. We propose to name this property as *silicodactyly*, as it describes the number of dactyls (from the Greek word meaning fingers) used by the silane to grab the surface: In Scheme 1, examples of di-dactyl (a) and tri-dactyl (b) hybrids are sketched. On the other hand, the catalytic head can be anchored to the surface through a variable number of alkoxysilane chains (*silicopodality*), giving rise to mono- or multi-podal systems: In Scheme 1, a dipodal structure (c) is exemplified and compared to the monopodal analogs (a) and (b).

In the present paper, we considered only the effect of *silicodactyly* on the conformation of the grafted alkoxysilane. As probe molecules, (3-mercaptopropyl)silanes with different numbers of methoxy groups on the silicon (the remaining valences being saturated by methyl groups) have been used. Mercapto-functional mesoporous silicas have received considerable attention as heavy-metal ion-trapping agents [14]. Moreover, the grafted thiol groups can be oxidized to provide sulfonic acids for applications in solid acid catalysis [15]. These molecules, where the -SH head represents the branching point for the possible organo catalyst, were grafted on an ordered mesoporous silica, MCM-41, and the hybrid surface so obtained was investigated by a combined experimental (XRD, TGA/DTG, ssNMR and FTIR) and computational approach. ^29^Si and ^13^C CPMAS NMR and FT-IR spectroscopy in controlled atmosphere were employed to establish whether the alkoxysilane derivatives undergo a complete hydrolysis and condensation with surface silanols. Moreover, the experimental techniques together with Molecular Dynamics calculations were used to describe the influence of the different grafting configurations (dactyly) on the organic chain configuration with respect to the surface.

## 2. Results and Discussion

### 2.1. Hybrid Structures

**Scheme 2:**
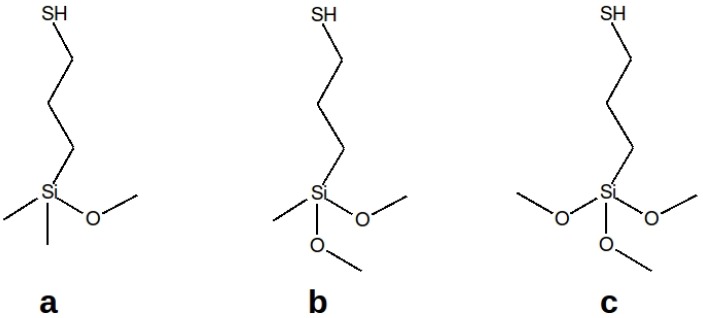
(**a**) 3-mercaptopropyl(methoxy)dimethylsilane, (**b**) 3-mercaptopropyl(dimethoxy)methylsilane and (**c**) 3-mercaptopropyl trimethoxysilane.

Three different 3-(mercaptopropyl)alkoxysilanes with a different number of hydrolysable moieties (one, two or three, Scheme 2), were grafted on an ordered mesoporous MCM-41. To avoid island-type grafting due to condensation and clustering of the silylation agents, the grafting was carried out using a clean inorganic surface in a non-polar solvent, like toluene. These reaction conditions favor uniform distributions of the organic moieties [16]. In Table 1 the acronyms of the hybrids are reported.

The MCM-41 ordered mesoporous structure has not been affected by the grafting procedure to obtain the hybrid materials as evidenced by XRD analysis (Figure S1 in the Supporting Information).

**Figure 1:**
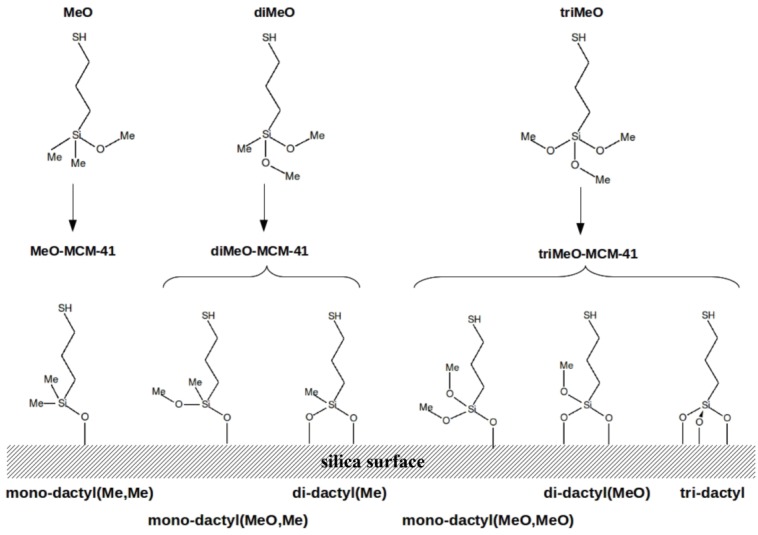
Hybrid systems possibly obtained by grafting the three (3-mercaptopropyl)alkoxysilanes on MCM-41 silica. The names in the lowest lines highlight the *dactyly* of the hybrid and the nature of the non-grafted dangling groups.

Depending on the number of methoxy groups which are actually hydrolyzed during the grafting, from the three mercapto precursors, six different hybrid structures can be obtained, as shown in Figure 1, where a naming scheme is also proposed to identify the grafted structures.

### 2.2. Thermogravimetric Analysis

Thermogravimetric analysis was performed in order to gain insight into the organic content, the thermal stability of the grafted mercapto silane and the hydrophobic/hydrophilic character of the hybrid materials (Figure S2 in the Supplementary Information). The first weight loss observed at around 80–150 °C can be associated to the removal of physisorbed water. At higher temperatures (150–550 °C), flat weight loss profiles of the hybrids changed into a rapidly declining profile corresponding to the decomposition of the organic silane. In particular, MeO-MCM-41 showed a weight loss due to the physisorbed water similar to plain MCM-41, while diMeO-MCM-41 and triMeO-MCM-41 lost a higher amount of water, meaning a lower hydrophilicity character of these latter functionalized surfaces. On the contrary, at high temperature, triMeO-MCM-41 lost a higher fraction of organic content with respect all the other hybrids. At higher temperature (550–800 °C), the weight loss of plain MCM-41 is due to silanol condensation forming siloxane bridges. The organic content, calculated from the weight loss in the 150–550 °C range, due to the decomposition of the grafted mercapto silane, is reported in Table 2. In addition, from the DTG curves (Figure S3 in the Supplementary Information), it is possible to observe that the temperature of weight loss due to the grafted mercapto silane for MeO-MCM-41 is slightly lower.

### 2.3. FTIR Analysis

**Figure 2:**
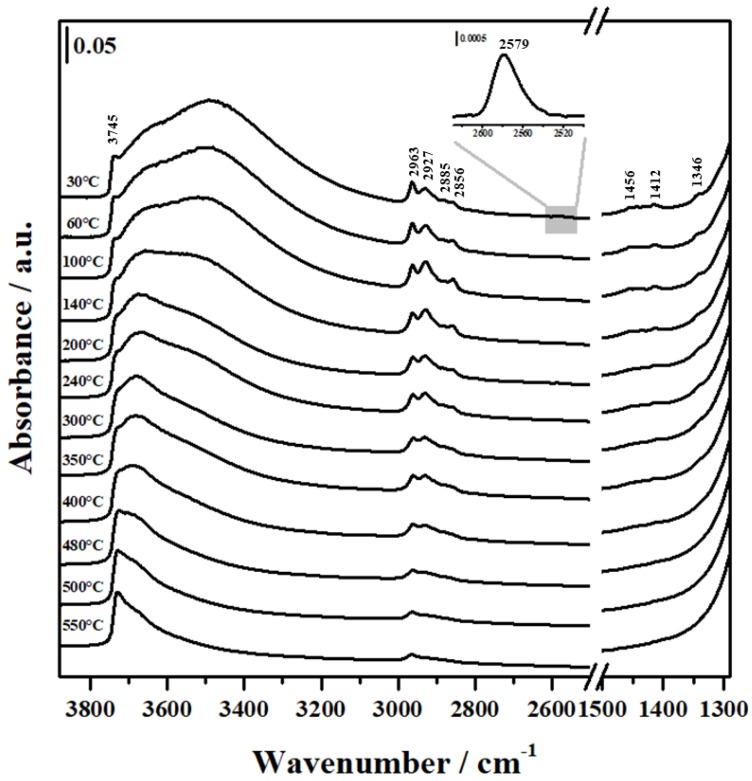
Variable temperature FTIR spectra of MeO-MCM-41.

**Figure 3:**
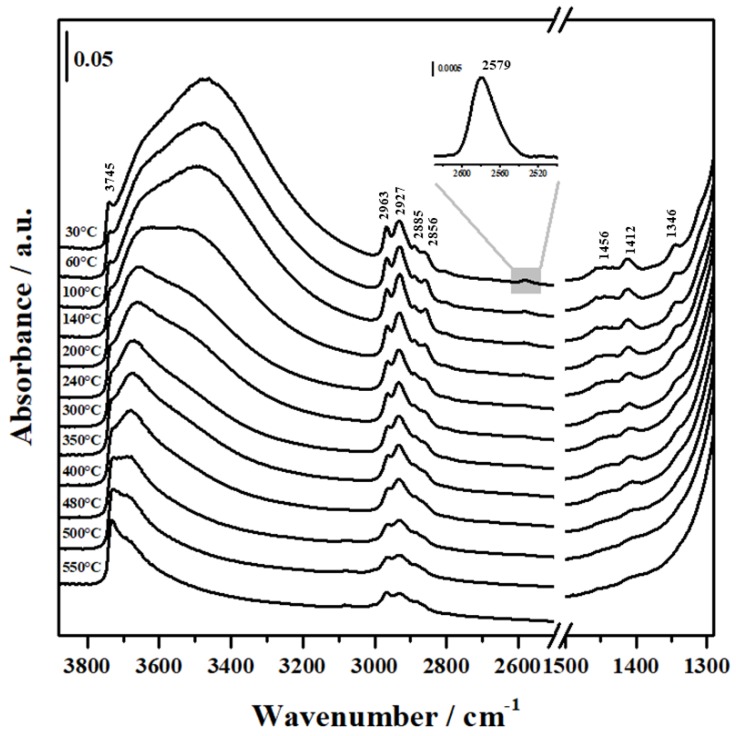
Variable temperature FTIR spectra of diMeO-MCM-41.

**Figure 4:**
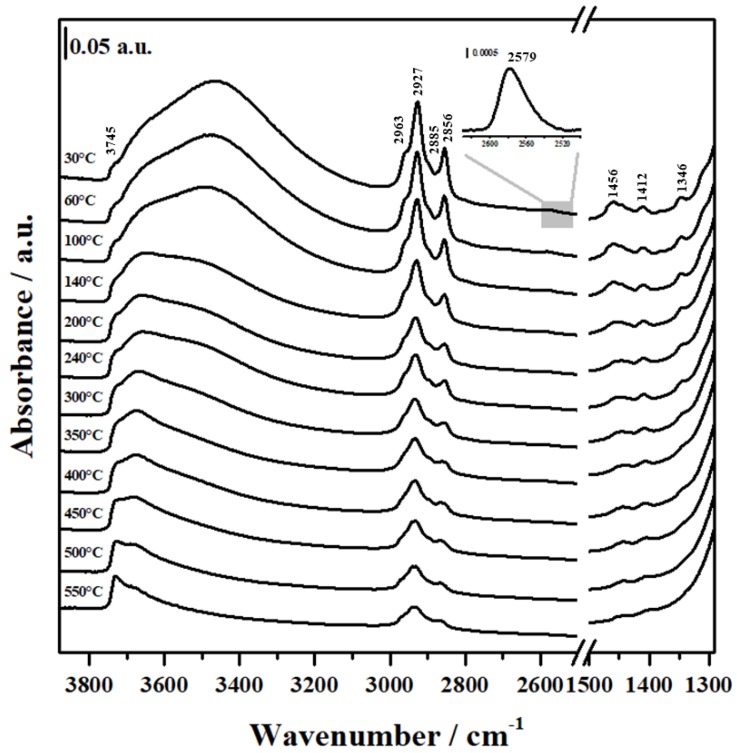
Variable temperature FTIR spectra of triMeO-MCM-41.

The influence on the thermal stability of mercaptopropyl chain grafted via one, two or three anchoring groups was investigated by FTIR spectroscopy experiments carried out at different temperatures (Figure 2, Figure 3 and Figure 4). The FTIR spectra of the three hybrids upon outgassing at 30 °C showed at high wavenumber, one weak band at 3745 cm^−1^ with a broad absorption between 3700 and 2500 cm^−1^. The weak band at 3745 cm^−1^ is related to residual isolated Si-OH and it is less intense in the triMeO-MCM-41 sample. The bands at 2963 and 2855 cm^−1^, due to the asymmetric and symmetric C-H stretching mode of CH_3_ groups, and the bands at 2927 and 2856 cm^−1^, due to the ν_as_ and ν_sym_ modes of CH_2_ groups in the propyl chain, confirm surface functionalization, whereas the broad absorption between 3700 and 2500 cm^−1^ evidences the presence of hydrogen-bonding interactions between surface groups [17]. The bands corresponding to the CH_2_ bending vibrations are also detected with low intensity in the range 1470–1400 cm^−1^. A weak signal is observed at 2579 cm^−1^ in all samples due to the S-H stretching mode of the thiol groups [18,19,20]. By increasing outgassing temperature, the organic fraction of the hybrids started to decompose at ca. 240 °C and the intensities of the FTIR bands, in the 3000–2800 cm^−1^ and 1470–1300 cm^−1^ ranges, decreased. In particular, FTIR spectra evidenced that the grafted mercapto-silane of MeO-MCM-41 hybrid decomposed almost totally at ca 400 °C while for the other hybrids, the organic fraction decomposed at temperature slightly higher than 400 °C, in agreement with TG analysis.

### 2.4. ss-NMR Analysis

**Figure 5:**
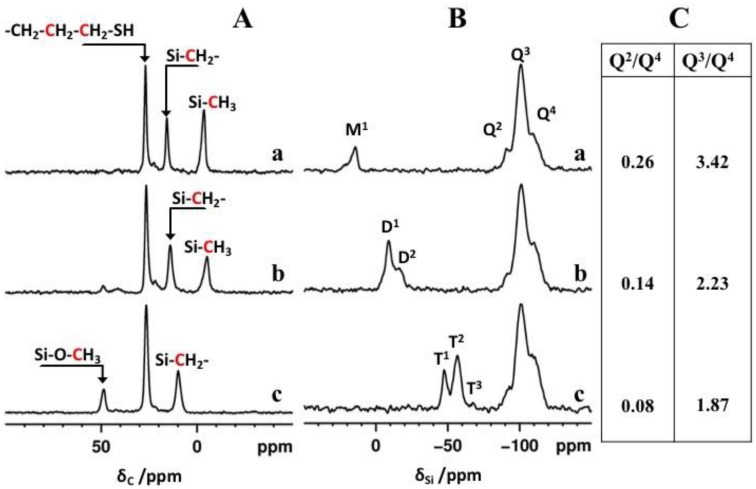
^13^C CPMAS NMR (**A**) and ^29^Si CPMAS NMR (**B**) of MeO-MCM-41 (curve a), diMeO-MCM-41 (curve b) and triMeO-MCM-41 (curve c). Area ratios derived from the deconvolution of ^29^Si CPMAS NMR data (**C**).

^13^C CPMAS NMR analysis (Figure 5A) was used to confirm the degree of reaction between alkoxy functions from organosilane reagents and silica support silanols. The resonance peaks at −3.6 and −5.2 ppm are due to the Si-**C**H_3_ dangling groups in MeO-MCM-41 (curve a) and diMeO-MCM-41 (curve b) hybrids, respectively. On the other hand, the Si-**C**H_2_ signal is present at 15.7 and 14 ppm in MeO-MCM-41 and diMeO-MCM-41 hybrids, respectively. Similarly, an up-field shifted peak at 9.8 ppm was observed in triMeO-MCM-41 (curve c) due to Si-**C**H_2_ groups. Finally, the other two methylene carbons (-CH_2_-**C**H_2_-**C**H_2_-SH) resonate at around 26.5 ppm in all the three hybrid materials. Resonance peaks at ca. 49 ppm are commonly ascribed to the residual non-hydrolyzed methoxy groups and are particularly evident in triMeO-MCM-41 (curve c) meaning that the presence of three alkoxy groups in the silane precursor will not ensure a complete grafting to three surface silanols [21,22].

The ^29^Si CPMAS NMR experiment (Figure 5B), which enhances, by magnetization transfer, the sensitivity and detection of the silicon atoms nearby to protons, allows a thorough characterization of the surface species grafted to the MCM-41 support. The crystallographically distinct Si sites are identified according to the commonly used Q^n^ and T^n^/D^n^/M^n^ notations [23]. The ^29^Si NMR chemical shifts for various Si sites are detected in the following ranges. Q^4^ (**Si**(OSi)_4_) silicon site at −110 ppm, Q^3^ (**Si**(OSi)_3_OH) site at −101 ppm and Q^2^ (**Si**(OSi)_2_(OH)_2_) site at −91 ppm. Similarly, the T silicon sites [T^3^ (R**Si**(OSi)_3_) at −67 ppm, T^2^ (R**Si**(OSi)_2_OH) sites at −57 ppm and T^1^ (R**Si**(OSi)(OH)_2_ sites at −48 ppm], the D silicon sites [D^2^ (R_2_**Si**(OSi)_2_) sites at −16 ppm and D^1^ (R_2_**Si**(OSi)(OH) sites at −9 ppm] and the M silicon site [M^1^ (R_3_**Si**(OSi)) site at +15 ppm] also exhibit characteristic chemical shifts.

In the organosilane grafting process onto silica support, the Q^3^ Si sites play a crucial role since they provide the Si-OH groups for silane grafting [24]. On the other hand, Q^4^ Si sites do not directly participate in the grafting process as they lack OH groups. Although the deconvolution data from the CPMAS experiments are not quantitative, a trend in the grafting process can be derived from the Q^2^/Q^4^ and Q^3^/Q^4^ ratios.

The monofunctional silane (curve a) grafted onto the MCM-41 shows a signal at ca. 15 ppm, the M signal, the difunctional silane (curve b) has signals at ca. at −9 and −16 ppm, due to D^1^ and D^2^ Si sites, respectively. Similarly, the trifunctional silane (curve c) give rise to signals in the −70 to −45 ppm range, associated to T silicon sites. TriMeO-MCM-41 (curve c) shows more intense T^2^ and T^1^ Si sites, from the reacted mercapto silane molecules, suggesting that the trifunctional silane has reacted in a bi- or mono-dentate manner, respectively. However, very weak signal due to T^3^ silicon site at around −68 ppm confirms the presence of a minor (if any) fraction of tri-dentate silane grafted to the MCM-41 surface. Nevertheless, the surface grafting mechanism may have occurred mostly through a bi-dentate chelation as has been confirmed by the higher intense peaks observed for T^2^ sites, along with the Si-O-CH_3_ signal in the ^13^C CPMAS NMR spectrum (Figure 5A, curve c). Borrego et al., have reported similar observations when they have employed (3-mercaptopropyl)trimethoxysilane as the silylation agent for grafting on mesoporous solids [24]. The hydrophobic nature of the methyl on the organosilanes might also impede the complete hydrolysis and multidentate anchoring on a hydrophilic surface like that of MCM-41 [25].

In all the hybrid samples, Q^4^, Q^3^ and Q^2^ signals associated to the MCM-41 silica support are also present, although with different intensities [24,26]. The Q^2^/Q^4^ and Q^3^/Q^4^ ratios decrease as we move from monodentate (MeO-MCM-41) to tridentate (triMeO-MCM-41) hybrids, essentially confirming the mono and/or bidentate (and, in a much less extent, tridentate) connectivity to the silica support surface (Figure 5C). Moreover, from the Q^2^/Q^4^ and Q^3^/Q^4^ ratios it is evident that Q^2^ sites show the highest decrease in triMeO-MCM-41 as geminal (Q^2^) OH will react preferably with tridentate silane. Such a reaction pathway involving Q^2^ site will lead to either monodentate or bidentate grafting as evidenced in triMeO-MCM-41.

**Figure 6:**
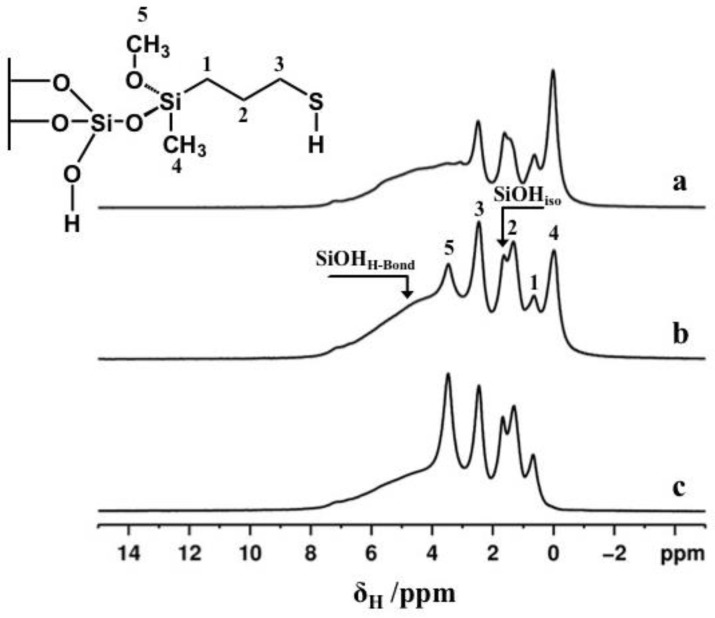
^1^H ECHO MAS NMR spectra of MeO-MCM-41 (curve a), diMeO-MCM-41 (curve b) and triMeO-MCM-41 (curve c).

Furthermore, ^1^H ECHO MAS NMR spectra (Figure 6) gave additional information on the proton environments and grafted organosilanes. The proton spectrum consists mainly of contributions from 3-mercaptopropyl groups, unhydrolyzed methoxy species, methyl species directly bonded to Si in organosilanes as well as silanols in either isolated or hydrogen-bonded state. The signals of -SH proton as well as of physisorbed water were difficult to distinguish in the ^1^H NMR spectra, however, their contributions will fall under the broad peak in the range 3–8 ppm. In addition, proton NMR data also reveal the strong presence of unhydrolyzed methoxy species in triMeO-MCM-41 whereas, moderate amounts in diMeO-MCM-41. Similarly, peaks due to isolated silanols were less intense in diMeO-MCM-41 and triMeO-MCM-41 hybrids, in agreement with FTIR experiments, emphasizing the multi-dentate bonding on the silica surface. Finally, the nature of thiol groups and its interaction with silanols and/or physisorbed water were further confirmed by preliminary analysis with variable contact time 2D proton-silicon heteronuclear correlation NMR experiments (data not shown for brevity).

### 2.5. Computational Modeling

Classical molecular dynamics (MD) calculations were used to obtain microscopic insights on the hybrid structures, with the computational parameters detailed in section “3. Materials and Methods”.

Six models, corresponding to the grafting schemes illustrated in Figure 1, were prepared by linking the suitable (3-mercaptopropyl)alkoxysilane to a periodic model of MCM-41 surface with a silanol density of 2.4 OH/nm^2^, in agreement with the experimental evaluation by TG analysis on plain MCM-41. The Cartesian coordinates of all the models are provided as Supporting Information. It is noteworthy that the tri-dactyl system could not be easily prepared on this kind of surface, unlike the di- and mono-dactyls, because of the scarcity of silanol groups in fit positions: Finally, the tri-dactyl hybrid was obtained by condensing two methoxy groups with geminal silanols, leading to a quite strained structure. This agrees with the ss-NMR findings described above, when only an exceedingly weak T^3^ peak could be detected in the triMeo-MCM-41 sample, suggesting that a more hydrophilic (i.e., with higher silanol density) silica surface should be used to obtain a higher concentration of tri-dactyl hybrids.

Then 1 ns MD runs at 298 K were performed for each model, after suitable thermalization cycles: During the simulations, the distance between sulphur atom and the silica surface was monitored, to measure the tendency of the organic chain to lie down on the surface.

**Figure 7:**
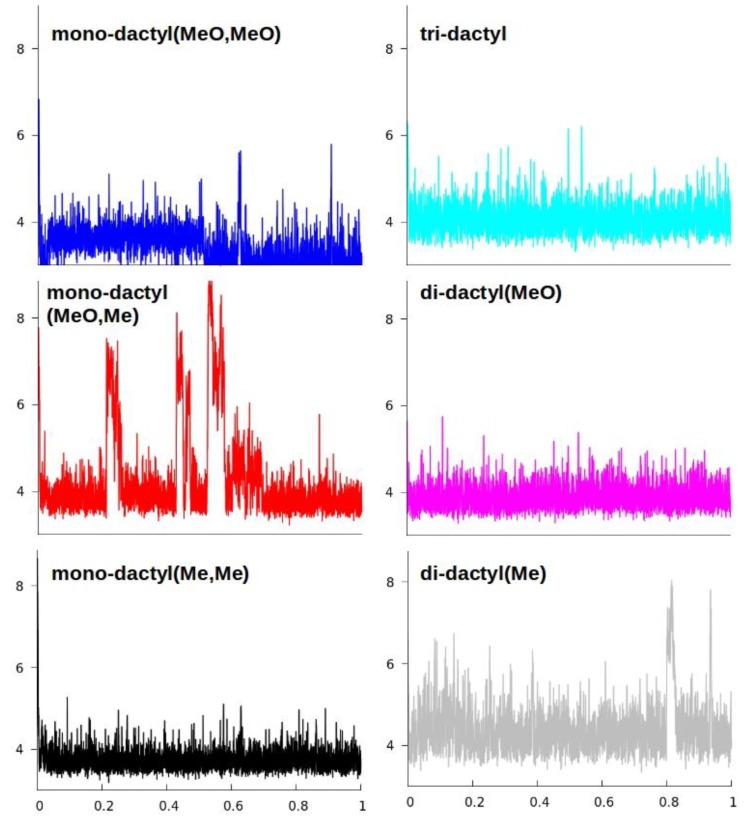
Time (ns) evolution of the sulphur-silica surface distance (Å) for the six hybrid models.

**Figure 8:**
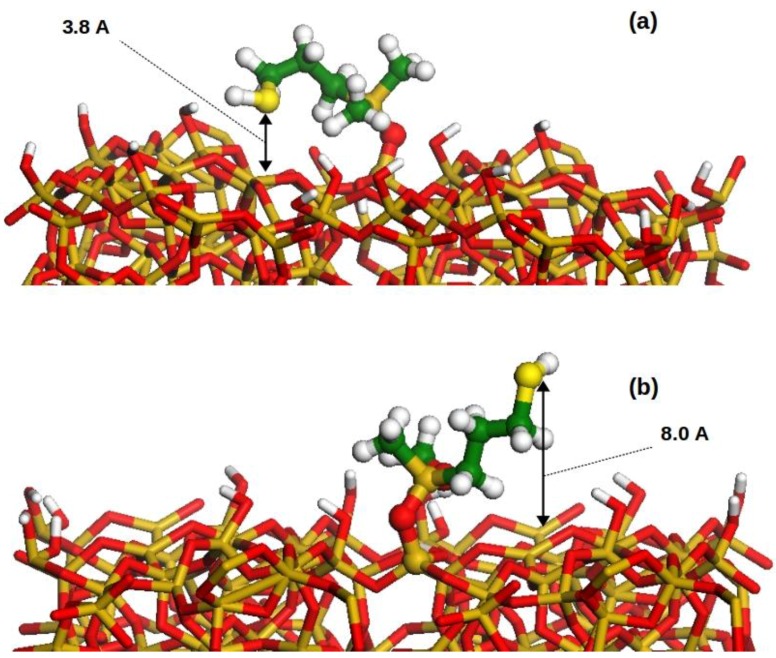
Snapshots of the MD simulations: (**a**) mono-dactyl(Me,Me) with the chain lying close the surface; (**b**) mono-dactyl(MeO,Me) with the chain extending further from the surface.

The time evolution of the sulphur-surface distance is presented for all the models in Figure 7, and the corresponding average distances are listed in Table 3. To facilitate the interpretation of the graphs, a couple of snapshots extracted from the dynamics are presented in Figure 8: We can see that a distance around 4 Å or below indicates a structure strongly bent and close to the surface, while in a standing, extended structure the sulphur-silica distance can reach 8–9 Å.

The results show that the silicodactyly *in se* has little effect on the average hybrid conformations: Tri-dactyl, di-dactyl(MeO) and two mono-dactyl structures lay close to the surface for all the simulation, with average distances in the 3.4–4.1 Å range; in particular mono-dactyl(MeO,MeO) spends half of the time very close to the silica, with distances around 3 Å. On the other hand, di-dactyl(Me) oscillates much more strongly, spending most of the time with a sulphur-surface distance around 4.5 Å; the most mobile structure is mono-dactyl(MeO,Me), the only one observed several times in extended conformation, though the average distance for this system (Table 3) is not the largest, but with a great standard deviation, accounting for the different conformations sampled in the dynamics.

The analysis of the MD simulation reveals that the largest contribution to the molecule-surface interaction energy comes from dispersion forces (included in the force field through a 6–12 Lennard-Jones term), with a minor but important contribution from H-bonds between silanols and chain oxygen and sulphur atoms. The latter effect is expected to become more sizeable with silica surfaces at higher silanol concentration. No relevant differences were detected between methyl and methoxy dangling groups, with respect to the tendency of the organic chain to lie down to the surface. Both groups show steric effects during MD runs, and sometimes methoxy oxygens are engaged in loose H-bonds with silanols, but this does not seem to correlate with the chain conformation.

## 3. Materials and Methods

### 3.1. General

MCM-41, methylmagnesium bromide and solvents were purchased from Sigma-Aldrich and employed as received. 3-Mercaptopropyltrimethoxysilane and 3-mercaptopropyl(dimethoxy) methylsilane were obtained from TCI Europe and used as received. 3-Mercaptopropyl(methoxy)dimethylsilane was prepared by a slight modification of a reported procedure [27].

### 3.2. 3-Mercaptopropyl(methoxy)dimethylsilane

In a 250 mL round bottomed flask under inert atmosphere (N_2_), 3-mercaptopropyltrimethoxysilane (8.0 mL, 43 mmol) was dissolved in dry THF (25 mL) and cooled to 0–5 °C with an ice bath. Methylmagnesium bromide (3M in diethyl ether, 43 mL, 129 mmol) was added dropwise at such a rate to maintain T < 10 °C. The olive-green mixture was stirred at 0–10 °C for 75 min during which the color gradually disappears. Dry methanol (25 mL) was then added (caution, foaming) and the milky-white suspension was diluted with a THF/diethyl ether (5mL/40mL) mixture. Filtration on a sintered glass frit under N_2_ atmosphere and evaporation of the solvents gave a liquid residue, fractionally distilled under vacuum to give the title product (4.9 g). Clear colorless liquid; yield, 69%; b.p. 70–75 °C (60 mmHg). ^1^H-NMR (CDCl_3_, 300 MHz, ppm): 3.39 (s, 3H, CH_3_O), 2.50 (q, 2H, J = 7.3 Hz, CH_2_S, 1.62 (m, 2H, C-CH_2_-C), 1.30 (t, 1H, J = 8.0 Hz, -SH), 0.66 (m, 2H, CH_2_-Si), 0.07 (s, 6H, CH_3_-Si). ^13^C-NMR (CDCl_3_, 75.6 MHz, ppm): 50.2 (CH_3_), 28.1 (CH_2_), 27.9 (CH_2_), 15.2 (CH_2_), −2.7 (CH_3_).

### 3.3. Preparation of the Hybrid Materials

The hybrid materials were prepared by post-synthesis grafting with 3-mercaptopropyl alkoxysilane. 0.3 g of MCM-41 (dried overnight at 100 °C) were suspended in 30 mL of anhydrous toluene. The suspension was heated at 120 °C under stirring. Then, 0.77 mmol of mercaptopropyl silane precursor was added dropwise and the mixture was allowed to reflux for 18 h. The reaction solution was cooled down, filtered and washed with toluene, ethanol and deionized water. The obtained white solid was cured in air at 80 °C overnight.

### 3.4. Characterization

Thermogravimetric analyses were carried out on a TA Q500 instrument (TA instrument, Sesto San Giovanni (MI), Italy), in nitrogen. The samples were heated from 35 to 800 °C at a heating rate of 10 °C/min under a gas flow rate of 60 mL/min.

X-ray powder diffraction (XRD) patterns were obtained using an ARL XTRA48 diffractometer (Thermo Fisher, Monza, Italy) with Cu Kα radiation (λ = 1.54062 Å).

FTIR spectra of self-supporting pellets at variable temperature were collected under vacuum conditions (residual pressure < 10^−4^ mbar) using a Bruker Equinox 55 spectrometer (Bruker, Milan, Italy) equipped with a pyroelectric detector (DTGS type) with a resolution of 4 cm^−1^. FTIR spectra were normalized with respect the pellet weight.

Solution state ^1^H and ^13^C NMR spectra were recorded with a Jeol Eclipse ECP300 spectrometer (Jeol Ltd. Tokyo, Japan) operating at 7.05T with operational frequencies for ^1^H, and ^13^C of 300.5 MHz and 75.6 MHz, respectively. Chemical shifts are reported in ppm with the protic impurities of the deuterated solvent as internal reference.

Solid-state NMR spectra were acquired on a Bruker Avance III 500 spectrometer and a wide bore 11.7 Tesla magnet with operational frequencies for ^1^H, ^29^Si and ^13^C of 500.13, 99.35 and 125.77 MHz, respectively. A 4 mm triple resonance probe with MAS was employed in all the experiments. The samples were packed on a Zirconia rotor and spun at a MAS rate between 10 and 15 kHz. The magnitude of radio frequency (RF) fields were 100 and 42 kHz for ^1^H and ^29^Si, respectively. The relaxation delay between accumulations was 1 s. For the ^13^C and ^29^Si Cross Polarization (CP) MAS experiments, the RF fields of 55 and 28 kHz were used for initial proton excitation and decoupling, respectively. During the CP period the ^1^H RF field was ramped using 100 increments, whereas the ^13^C/^29^Si RF fields were maintained at a constant level. During the acquisition, the protons are decoupled from the carbons/silicons by using a two-pulse phase-modulated (TPPM) decoupling scheme. A moderate ramped RF field of 62 kHz was used for spin locking, while the carbon/silicon RF field was matched to obtain optimal signal and the CP contact time of 2 ms were used. All chemical shifts are reported using δ scale and are externally referenced to tetramethylsilane (TMS) at 0 ppm.

### 3.5. Computational Details

#### 3.5.1. Design of Models

The structures of mercapto-propyl alkoxysilane derivatives are grafted on silica surface (slab) with density of 2.4 OH/nm^2^. The atomic structure of the slab is taken from model discussed e.g., by Ugliengo et al. [28]. The layer thickness is 13.96 Å. The horizontal dimensions of the simulation box are 25.34 × 26.55 Å^2^, while the vertical dimension is 50.00 Å. Periodic boundary conditions in all dimensions are used. One example of simulation box is reported in Figure S4 in the Supporting Information.

#### 3.5.2. General Settings of Molecular Dynamics

Starting configurations of the molecular dynamics are obtained performing partial energy minimization to adjust atom coordinates in the box: Only atomic positions of mercapto-propyl alkoxysilane derivatives and the silicon and oxygen atoms of slab directly bonded to these have been minimized. The remaining atoms are kept fixed.

We used Conjugate Gradients algorithm specifying an energy tolerance of 1.0 × 10^−3^ kcal/mol, and force tolerance of 0.5 kcal/mol/Å.

QEq charge equilibration method is used to generate atomic partial charge specifying a convergence limit of 1.0 × 10^−6^ e, using the ‘Qeq’ package implemented in Material Studio suite [29]. The Coulombic interactions are computed using standard Ewald summation with accuracy of 1.0 × 10^−6^ kcal/mol.

Van der Walls interaction are calculated as sum of 12–6 Lennard–Jones (LJ) using a cutoff 20 Å; σ and ε parameters have been extracted from UFF force field [30] and their amount are calculated based on the Lorentz-Berthold relationship.

Molecular dynamics simulations are performed in canonical (n,V,T) ensemble in vacuum at 298K (using Langevin thermostat to maintain a constant temperature) [31], for 1 nanosecond using the LAMMPS simulation package [32].

## 4. Conclusions

Mercapotopropylsilanes, with 1 to 3 hydrolyzable groups, have been successfully grafted on ordered mesoporous MCM-41 silica. The number of siloxane bridges that the mercaptopropylsilanes can form with the surface silanols of the inorganic support are strictly correlated with the number of hydrolysable groups of the organic moiety and the silanol concentration of the support. We named this property, that describes the number of fingers (*dactyls*) used by the silane to grab the surface, *silicodactyly*. A combined experimental (TGA, FT-IR and ss-NMR) and computational (MD) study has clarified the different hybrid structures that can be formed, and their conformations, along with the interactions of organic and inorganic parts in the hybrid materials. This combined approach has enlightened the influence of *silicodactyly* in the designing and engineering hybrid catalysts with accessible active organic sites. The computational studies clearly predict that the organic catalytic site can stick on the silica surface, and this relevant aspect should be primarily taken into account when designing an active hybrid catalyst.

## Figures and Tables

**Table 1:** Acronyms of the hybrid systems.

Acronyms	Mercapto Precursor Grafted on MCM-41
MeO-MCM-41	3-mercaptopropyl(methoxy)dimethylsilane
diMeO-MCM-41	3-mercaptopropyl(dimethoxy)methylsilane
triMeO-MCM-41	3-mercaptopropyl(trimethoxy)silane

**Table 2:** Weight loss (%) due to water and organic content calculated from TGA analysis.

Samples	Δwt% Due to H_2_O ^a^	Δwt% Due to Mercapto Silane ^b^
MCM-41	1.25	-
MeO-MCM-41	1.13	6.30
diMeO-MCM-41	0.45	8.20
triMeO-MCM-41	0.74	8.90

^a^ Physisorbed H_2_O calculated from 30 °C to 150 °C. ^b^ Calculated from 150 °C to 550 °C.

**Table 3:** Average sulphur-silica distance and standard deviation (Å) during the MD simulations of the hybrid materials.

Hybrid Structures	Average Distance (±stdev)/Å
tri-dactyl	4.09 (±0.35)
di-dactyl(MeO)	3.90 (±0.29)
di-dactyl(Me)	4.42 (±0.63)
mono-dactyl(MeO,MeO)	3.42 (±0.44)
mono-dactyl(MeO,Me)	4.29 (±1.05)
mono-dactyl(Me,Me)	3.75 (±0.03)

## Supplementary Materials

The Supplementary Materials are available online, Figure S1: The XRD pattern of MeO-MCM-41, diMeO-MCM-41 and triMeO-MCM-41. Figures S2 and S3: TGA and DTG curves of plain MCM-41, MeO-MCM-41, diMeO-MCM-41 and triMeO-MCM-41. Figure S4: The simulation box contains one thiol-propyl alkoxysilane derivative grafted on slab silica and periodic boundary conditions in all direction are used.
