# Supplementary material for: Influence of Silicodactyly in the Preparation of Hybrid Materials
Source: Molecules. 2019 Feb 28;24(5):848. doi: 10.3390/molecules24050848 (PMC6429323; doi:10.3390/molecules24050848)
Supplement: Supplementary file 1 [file molecules-24-00848-s001.pdf]

molecules
MDPI

SUPPORTING INFORMATION

Influence of silicodactyly in the preparation of hybrid materials

Chiara Ivaldi[1], Ivana Miletto[1], Geo Paul[1], Giovanni B. Giovenzana[2], Alberto Fraccarollo[1], Maurizio Cossi[1], Leonardo Marchese[1] and Enrica Gianotti[1,*]

[1] Dipartimento di Scienze e Innovazione Tecnologica, Università del Piemonte Orientale, V. T. Michel 11, I-15100 Alessandria, Italy.
[2] Dipartimento di Scienze del Farmaco, Università del Piemonte Orientale, Largo Donegani 2/3, I-28100 Novara, Italy
* Correspondence: enrica.gianotti@uniupo.it; Tel.: +39-0131-360-251

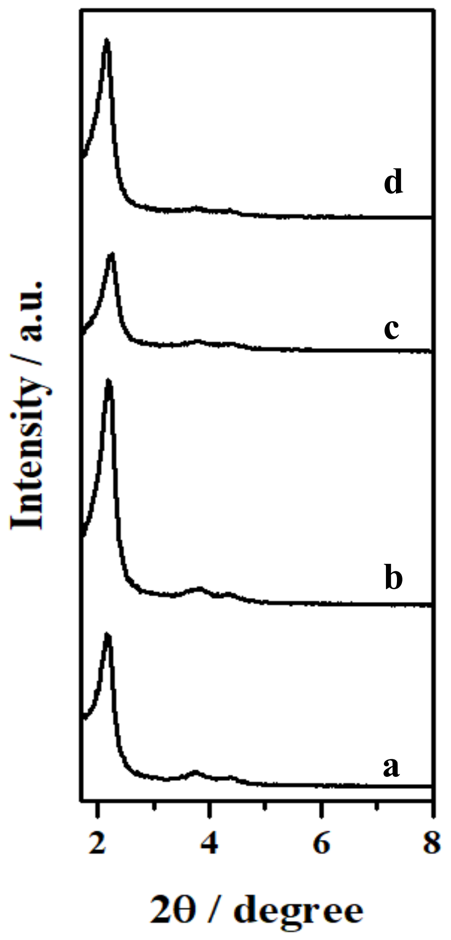

Figure S1 The XRD pattern of plain MCM-41 (curve a), MeO-MCM-41 (curve b), diMeO-MCM-41 (curve c) and triMeO-MCM-41 (curve d).

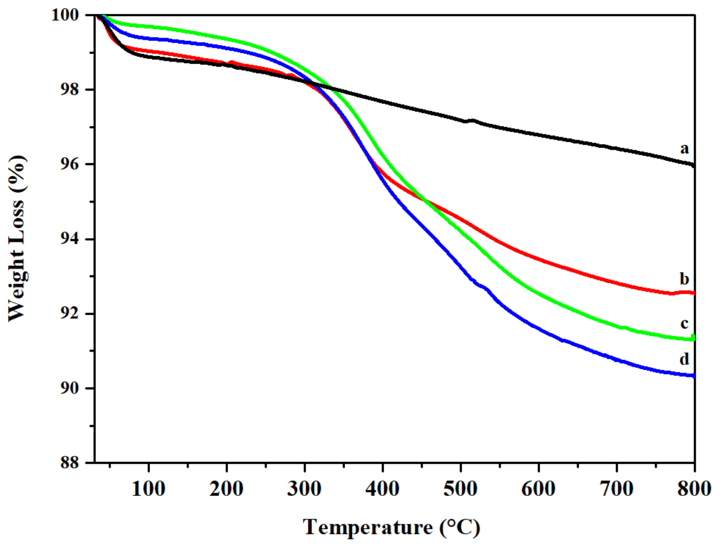

Figure S2. TGA curves of plain MCM-41 (a), MeO-MCM-41(b), diMeO-MCM-41 (c) and triMeO-MCM-41 (d).

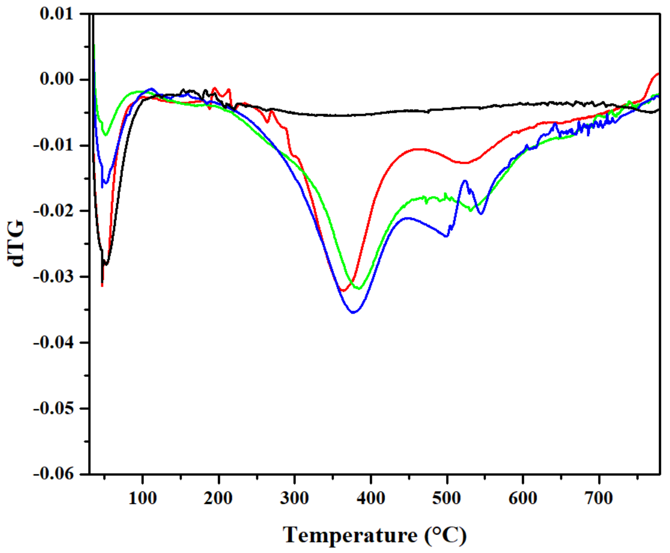

Figure S3. DTG curves of plain MCM-41 (black), MeO-MCM-41 (red), diMeO-MCM-41 (green) and triMeO-MCM-41 (blue).

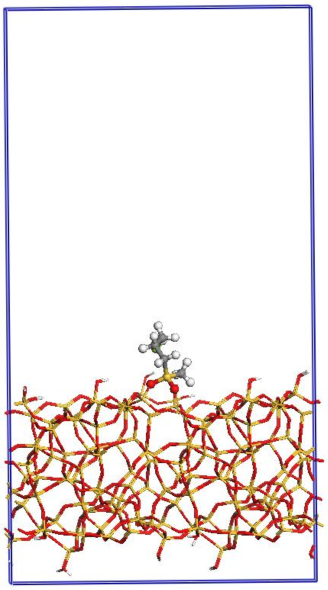

Figure S4. The simulation box contains one mercapto-propyl alkoxysilane derivative grafted on slab silica and periodic boundary conditions in all direction are used.
